# Destruction of Lymphoid Organ Architecture and Hepatitis Caused by CD4^+^ T Cells

**DOI:** 10.1371/journal.pone.0024772

**Published:** 2011-09-23

**Authors:** Matthias S. Matter, Tamara Hilmenyuk, Christina Claus, Romina Marone, Christian Schürch, Marianne Tinguely, Luigi Terracciano, Sanjiv A. Luther, Adrian F. Ochsenbein

**Affiliations:** 1 Tumor Immunology, Department of Clinical Research, University of Bern, Bern, Switzerland; 2 Department of Pathology, University of Basel, Basel, Switzerland; 3 Department of Biomedicine, Institute of Biochemistry and Genetics, University of Basel, Basel, Switzerland; 4 Institute of Surgical Pathology, University Hospital of Zurich, Zurich, Switzerland; 5 Department of Biochemistry, University of Lausanne, Epalinges, Switzerland; 6 Institute for Medical Oncology, University of Bern, Inselspital, Bern, Switzerland; MRC National Institute for Medical Research, United Kingdom

## Abstract

Immune responses have the important function of host defense and protection against pathogens. However, the immune response also causes inflammation and host tissue injury, termed immunopathology. For example, hepatitis B and C virus infection in humans cause immunopathological sequel with destruction of liver cells by the host's own immune response. Similarly, after infection with lymphocytic choriomeningitis virus (LCMV) in mice, the adaptive immune response causes liver cell damage, choriomeningitis and destruction of lymphoid organ architecture. The immunopathological sequel during LCMV infection has been attributed to cytotoxic CD8^+^ T cells. However, we now show that during LCMV infection CD4^+^ T cells selectively induced the destruction of splenic marginal zone and caused liver cell damage with elevated serum alanin-transferase (ALT) levels. The destruction of the splenic marginal zone by CD4^+^ T cells included the reduction of marginal zone B cells, marginal zone macrophages and marginal zone metallophilic macrophages. Functionally, this resulted in an impaired production of neutralizing antibodies against LCMV. Furthermore, CD4^+^ T cells reduced B cells with an IgM^high^IgD^low^ phenotype (transitional stage 1 and 2, marginal zone B cells), whereas other B cell subtypes such as follicular type 1 and 2 and germinal center/memory B cells were not affected. Adoptive transfer of CD4^+^ T cells lacking different important effector cytokines and cytolytic pathways such as IFNγ, TNFα, perforin and Fas-FasL interaction did reveal that these cytolytic pathways are redundant in the induction of immunopathological sequel in spleen. In conclusion, our results define an important role of CD4^+^ T cells in the induction of immunopathology in liver and spleen. This includes the CD4^+^ T cell mediated destruction of the splenic marginal zone with consecutively impaired protective neutralizing antibody responses.

## Introduction

Immune protection against pathogens must be balanced co-evolutionarily against lethal damage by immune responses. The process of host tissue destruction by the own immune system is termed immunopathology. Immunopathological sequel occurs during important infections in humans and mice. For example, after infection with hepatitis B and C virus in men, the T cell response causes liver cell damage. Similarly, the T cell response against lymphocytic choriomeningitis virus (LCMV) leads to destruction of secondary lymphoid organs, hepatic damage and choriomeningitis [1], [2].

Secondary lymphoid organs are highly organized structures, where B and T cells are localized to specialized zones. In contrast to lymph nodes, the lymphoid compartment of the spleen contains an additional structure called marginal zone, which consists of marginal zone macrophages, marginal zone metallophilic macrophages and marginal zone B cells [3]. Non-hematopoietic stromal cells orchestrate the structure of secondary lymphoid organs by expression of chemokines such as CCL19, CCL21, and CXCL13. The integrity of lymphoid organ architecture provides the basis for an optimal adaptive immune response. Mice with disturbed lymphoid organ architecture such as alymphoplastic (aly/aly), LTβR^−/−^, LTβ^−/−^, LTα^−/−^ and TNFα^−/−^ mice have defects in the adaptive immune response of varying degree [4]. In addition to LCMV, destruction of lymphoid architecture is caused by several pathogens such as HIV [5], *Plasmodium falciparum* (malaria) [6] and Lassa virus [7] in men and *Leishmania donovani* [8], *Borrelia crocidurae* (relapsing fever) [9] and murine cytomegalovirus [10] in mice. The destruction of secondary lymphoid organs is therefore an effective strategy employed by pathogens to suppress the host's immune system. The mechanisms of lymphoid organ architecture destruction are poorly defined in most of these infections, yet cytotoxic effects of the host's immune system may play a central role.

Immunopathology after LCMV infection has generally been attributed to cytotoxic CD8^+^ T cells, because CD8^+^ T cell depletion prevented the destruction of lymphoid organ architecture and hepatic damage [1], [11], [12]. However, in the absence of CD8^+^ T cells, viral load remains at high titers until approximately 40 days after infection when LCMV-specific neutralizing antibodies are mounted [13]. Importantly, the high virus load leads to functional inactivation of CD4^+^ T cells. The functional inactivation of CD4^+^ T cells is most likely a result of continued triggering by high viral load in combination with other parameters such as inflammatory cytokines, and expression of inhibitory receptors or pro-apoptotic molecules [14]. This is a relevant limitation of the CD8^+^ T cell-depletion experiments, and therefore a role of CD4^+^ T cells in the induction of immunopathology during LCMV-infection cannot be excluded.

Here, we studied the role of CD4^+^ T cells in the destruction of splenic architecture and hepatic damage during LCMV infection. To restore CD4^+^ T cell function, CD8-depleted BL/6 mice were adoptively transferred with LCMV-immune CD4^+^ T cells or LCMV-GP61-specific T cell receptor transgenic (SMARTA [15]) CD4^+^ T cells. We found that functional CD4^+^ T cells selectively destroy the splenic marginal zone, reduce protective LCMV-neutralizing antibodies and exert liver cell damage. Therefore, our results define an important role of CD4^+^ T cells in the induction of immunopathology in spleen and liver after LCMV infection.

## Results

### Adoptive transfer of LCMV-specific CD4^+^ T cells to CD8^+^ T cell-depleted mice rescues CD4^+^ T cell function in LCMV infection

**Figure 1:**
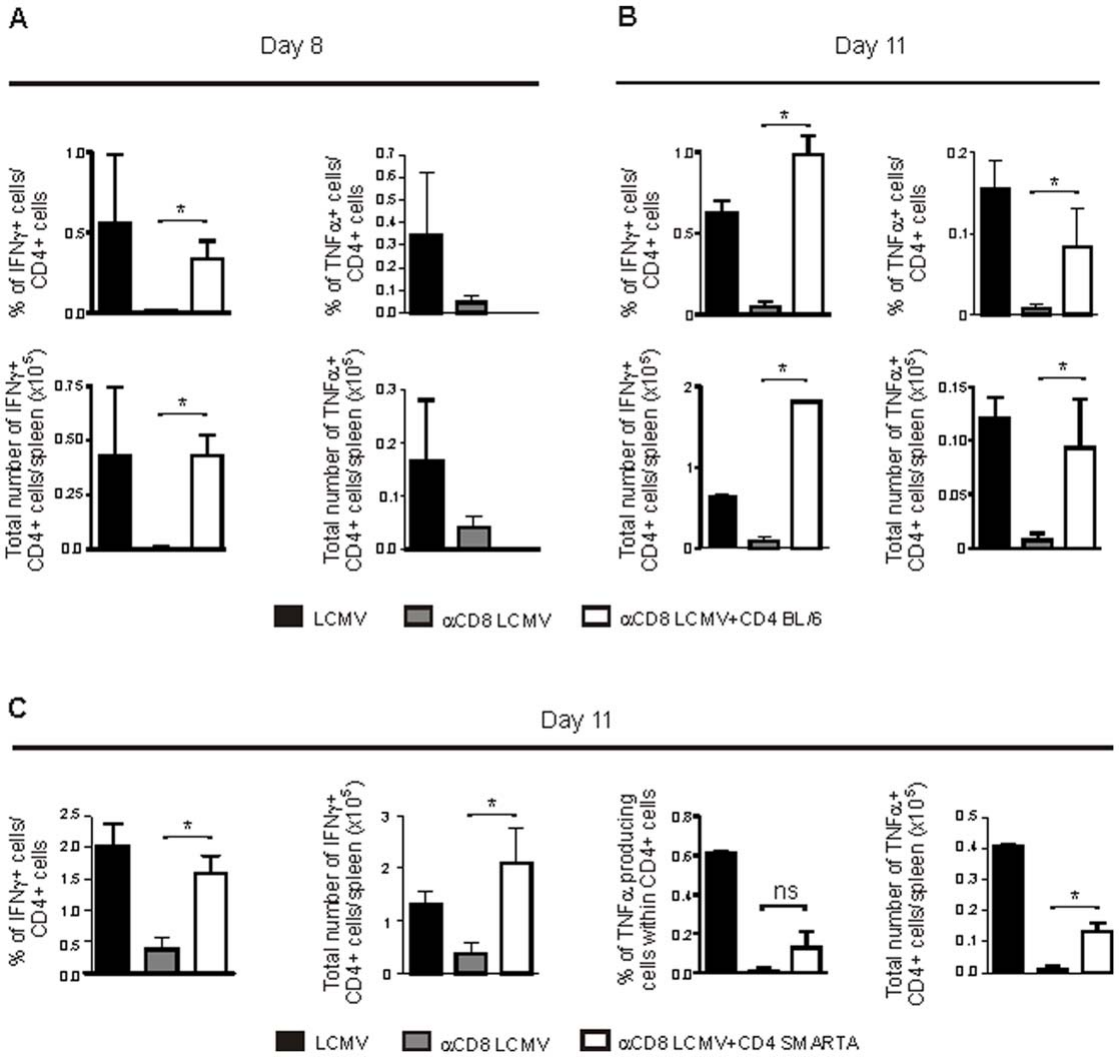
Model to study LCMV-specific CD4^+^ T cell responses in the absence of CD8^+^ T cells. (**A,B**) BL/6 mice, BL/6 mice depleted of CD8^+^ T cells and BL/6 mice depleted of CD8^+^ T cells receiving 1.5×10^7^ purified LCMV-immune CD4^+^ T cells were infected with LCMV. On day 8 (**A**) and 11 (**B**) after infection, CD4^+^ splenocytes were restimulated *in vitro* for 5 h with GP61 and analyzed for intracellular IFNγ and TNFα production by flow cytometry. Mean ± SEM (error bars) is shown. For A n = 3 animals per group, for (B) n = 2–3 animals per group, representative of two independent experiments. (**C**) BL/6 mice, BL/6 mice depleted of CD8^+^ T cells and BL/6 mice depleted of CD8^+^ T cells receiving 10^7^ naive SMARTA splenocytes were infected with LCMV. On day 11 after infection, CD4^+^ splenocytes were restimulated *in vitro* for 5 h with GP61 and analyzed for intracellular IFNγ and TNFα production by flow cytometry. Mean ± SEM (error bars) of 2–3 animals per group is shown.

BL/6 mice clear LCMV-WE doses of up to 10^6^ pfu within two weeks after infection below the detection limit of conventional plaque forming assays [16]. Control of LCMV is primarily mediated by cytotoxic CD8^+^ T cells and depletion of CD8^+^ T cells leads to high viremia until antibodies are mounted approximately 40 days after infection [13]. To analyze the function of LCMV-specific CD4^+^ T cells in the absence of CD8^+^ T cells and high viremia, BL/6 mice were depleted of CD8^+^ T cells by monoclonal antibody and infected with LCMV. As shown previously [13], CD8^+^ T cell depletion resulted in a drastic reduction of the relative and absolute number of LCMV-specific CD4^+^ T cells producing IFNγ and TNFα on day 8 and 11 after infection (Fig. 1A,B). For example on day 11, the mean percentage of CD4^+^ T cell producing IFNγ after restimulation with GP61 decreased significantly from 0.63% to 0.05% and the mean absolute number of IFNγ-producing CD4^+^ T cells dropped significantly from 0.65×10^5^ to 0.085×10^5^.

In order to obtain functional LCMV-specific CD4^+^ T cells in CD8-depleted BL/6 mice, the following experiment was performed: First, naïve BL/6 mice were depleted of CD8^+^ T cells by monoclonal antibody. On the same day, splenocytes of LCMV-infected BL/6 mice (17 days post infection) were harvested, purified for CD4^+^ T cells by MACS and adoptively transferred i.v. to these naïve CD8-depleted BL/6 mice. Each mouse received 1.5×10^7^ purified CD4^+^ T cells, which contained about 2.5×10^5^ IFNγ-producing LCMV GP61-specific CD4^+^ T cells (data not shown). Subsequently, mice were infected with LCMV (10^6^ pfu). On day 8 and 11 after infection CD4^+^ T cell function was analyzed in spleen. The transfer of purified LCMV-immune CD4^+^ T cells to CD8-depleted mice largely restored the relative and absolute number of LCMV-specific CD4^+^ T cells producing IFNγ at day 8 and 11 after infection (Fig. 1A,B). Furthermore, it increased the relative and absolute numbers of LCMV-specific CD4^+^ T cells producing TNFα on day 11, but not on day 8 after infection (Fig. 1A,B). In analogy, the adoptive transfer of naive LCMV-GP61-specific CD4^+^ T cells (transgenic SMARTA [15] cells) to CD8-depleted BL/6 mice increased the number of IFNγ-producing and to a lesser extent of TNFα-producing LCMV-specific CD4^+^ T cells on day 11 after LCMV infection (Fig. 1C). Importantly, CD4^+^ T cell function after adoptive transfer to CD8-depleted BL/6 mice was comparable to that observed in CD8^+^ T cell competent BL/6 mice. Therefore, this experimental set-up allows us to analyze the role of CD4^+^ T cells in the induction of immunopathology at a physiological frequency in the absence of CD8^+^ T cells.

**Figure 2:**
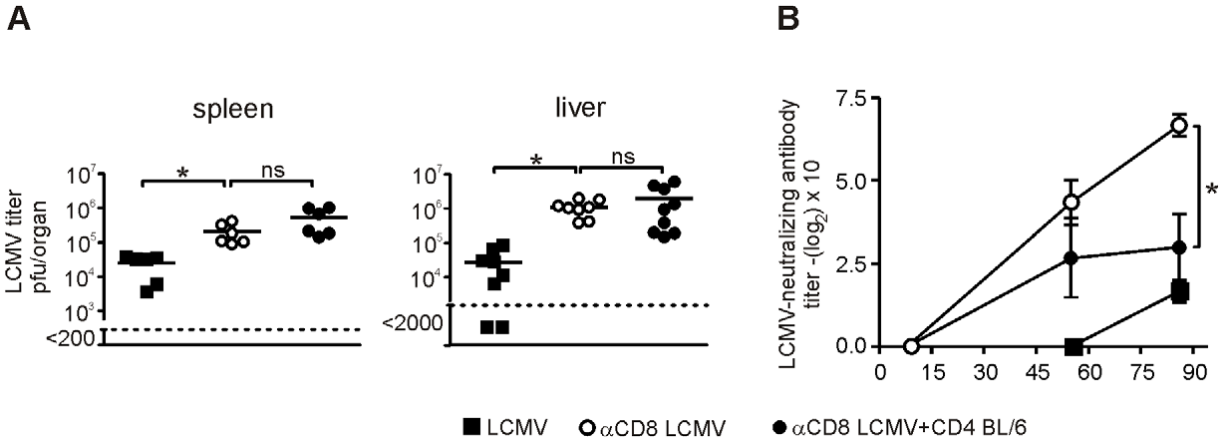
Virus control by CD8^+^ and CD4^+^ T cells. (**A**) BL/6 mice, BL/6 mice depleted of CD8^+^ T cells and BL/6 mice depleted of CD8^+^ T cells receiving 1.5×10^7^ purified LCM-immune CD4^+^ T cells were infected with LCMV. On day 11 after infection LCMV virus titer was determined in spleen and liver by plaque forming assay. Each symbol represents one mouse, data was pooled from 3 independent experiments. Horizontal bar indicates the mean. (**B**) BL/6 mice, BL/6 mice depleted of CD8^+^ T cells and BL/6 mice depleted of CD8^+^ T cells receiving 1.5×10^7^ purified LCMV-immune CD4^+^ T cells were infected with LCMV. Neutralizing antibodies against LCMV in the serum was analyzed on day 8, 55 and 86 after infection. Symbols represent mean ± SEM (n = 3).

Adoptive transfer of LCMV-immune CD4^+^ T cells to CD8-depleted BL/6 mice did not reduce the viral load in spleen or liver on day 11 after infection (Fig. 2A). In contrast, LCMV elimination was dependent on CD8^+^ T cells. Similarly, adoptive transfer of naive SMARTA cells did not influence viral elimination in spleen (data not shown).

### Reduced neutralizing antibody titers after transfer of activated CD4^+^ T cells

To test the consequence of adoptively transferred CD4^+^ T cells on antibody production, we measured LCMV-specific neutralizing antibodies under the same experimental conditions as described for Figure 1A and B. LCMV-specific neutralizing antibodies in BL/6 mice evolved late, 60–80 days after infection, and were of low magnitude (Fig. 2B). In contrast, BL/6 mice depleted of CD8^+^ T cells produced LCMV-specific neutralizing antibodies earlier and at higher titers (Fig. 2B). Interestingly, the adoptive transfer of LCMV-immune CD4^+^ T cells to CD8-depleted BL/6 mice significantly reduced neutralizing antibody titers. Therefore, CD4^+^ T cells impede the production of LCMV-specific neutralizing antibodies.

### Intact splenic T and B cell regions after transfer of activated CD4^+^ T cells

**Figure 3:**
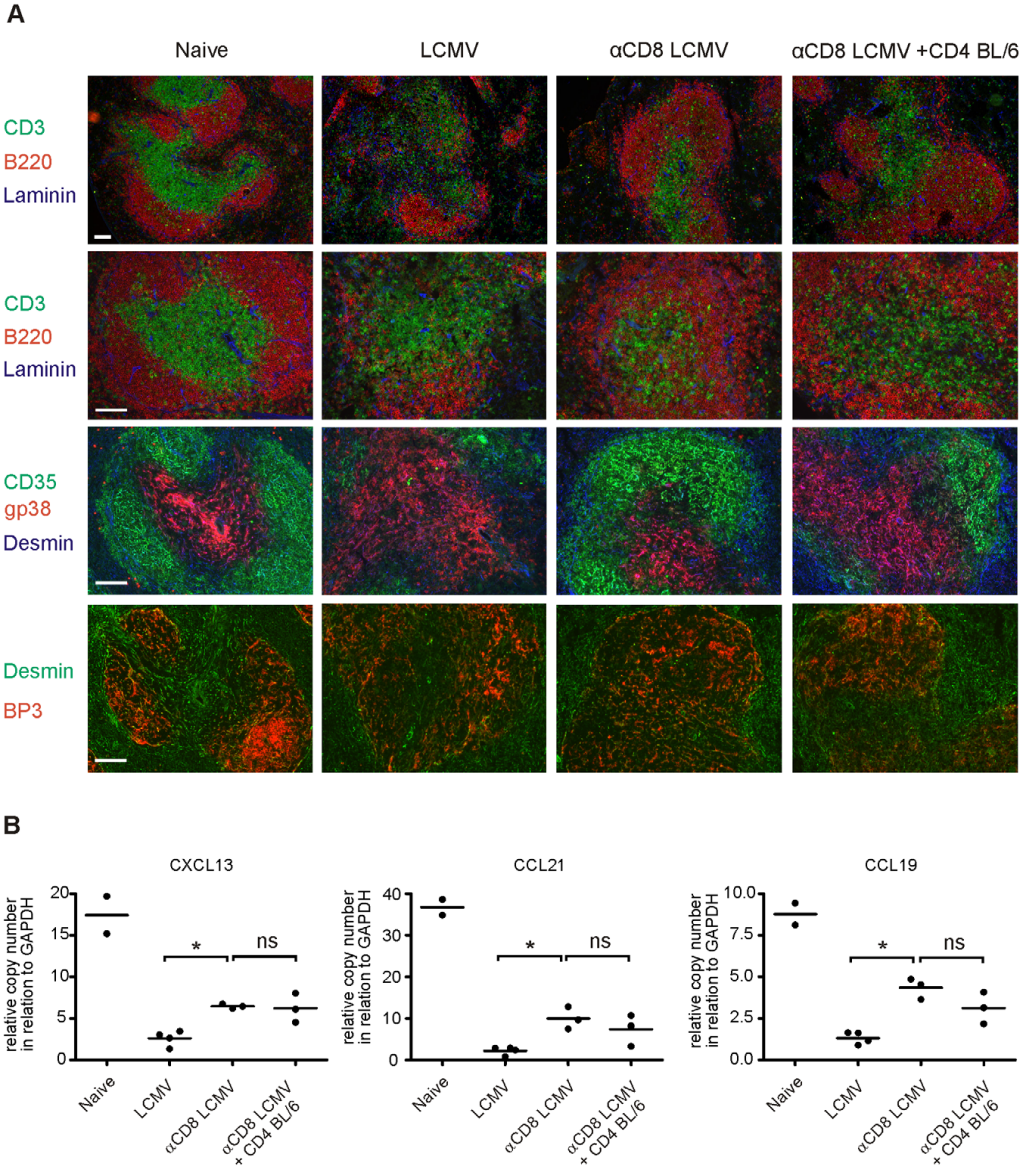
Immunofluorescence analysis of B and T cell regions. (**A**) BL/6 mice, BL/6 mice depleted of CD8^+^ T cells, and BL/6 mice depleted of CD8^+^ T cells receiving 1.5×10^7^ purified LCMV-immune CD4^+^ T cells were infected with LCMV. On day 11 after infection spleen sections were stained for CD3 (T cells; green), B220 (B cells; red), Laminin (blue), or CD35 (FDCs; green), gp38 (FRCs; red), Desmin (stromal cells, blue), or BP-3 (follicular stromal cells; red) and Desmin (green). One representative section of 3 mice per group is shown. BL/6 naïve mice served as control. Size bar represents 100 µm. The top row provides an overview with the rows below showing a higher magnification and consecutive sections. (**B**) The same experimental groups as in (A) were analyzed for the expression of CXCL13, CCL21, and CCL19 in the spleen by rtPCR. Each sample was analyzed in duplicate. Each symbol represents the mean of one animal. Horizontal bars indicate mean.

Reduced production of LCMV-specific neutralizing antibodies has been linked to the destruction of splenic architecture [1], [16]. Therefore, we performed immunofluorescence microscopy analysis on histological slides 11 days after LCMV infection from spleens of BL/6, CD8-depleted BL/6 and CD8-depleted BL/6 mice receiving LCMV-immune CD4^+^ T cells (Fig. 3A). LCMV-infected BL/6 mice had disrupted white pulp structures, as shown by staining for CD3^+^ T cells and B220^+^ B cells. In addition, follicular dendritic cells (FDCs, CD35^+^BP3^+^Desmin^+^), representing stromal cells of the B cell zone were strongly reduced. The networks of fibroblastic reticular cells (FRCs, gp38^+^desmin^+^), representing stromal cells of the T cell zone, were less reticular in LCMV-infected BL/6 mice. In contrast, in CD8-depleted BL/6 mice, B cell regions including FDC networks were preserved, while T cell regions including FRC networks were still partially disrupted. Interestingly, adoptive transfer of LCMV-immune CD4^+^ T cells to CD8-depleted BL/6 mice did not lead to a further disorganization of B and T cell regions including FDC and FRC networks when compared to CD8-depleted mice.

In a next experiment, the expression of the chemokines CXCL13, CCL19, and CCL21 was analyzed by quantitative PCR in the spleen (Fig. 3B). These chemokines are produced by FDCs and FRCs and are important for B and T zone compartmentalization [3]. LCMV-infected BL/6 mice showed a vigorous reduction of chemokine expression 11 days after infection [11], which was less pronounced in CD8-depleted mice [17]. Importantly, CD8-depleted mice receiving LCMV-immune CD4^+^ T cells did not show a statistically significant difference of chemokine levels to CD8-depleted mice. In accordance, immunofluorescence microscopy analysis of CXCL13 and CCL21 protein expression showed a strong reduction in LCMV infected BL/6 mice (Fig. S1). Both chemokines were less reduced in CD8-depleted mice without a clear difference to CD8-depleted mice receiving LCMV-immune CD4^+^ T cells. Taken together, these experiments revealed that the adoptive transfer of CD4^+^ T cells to CD8-depleted mice neither contributed to a substantial destruction of the splenic white pulp nor to a reduction of CCL19, CCL21, and CXCL13 expression.

### Destruction of the splenic marginal zone by CD4^+^ T cells

**Figure 4:**
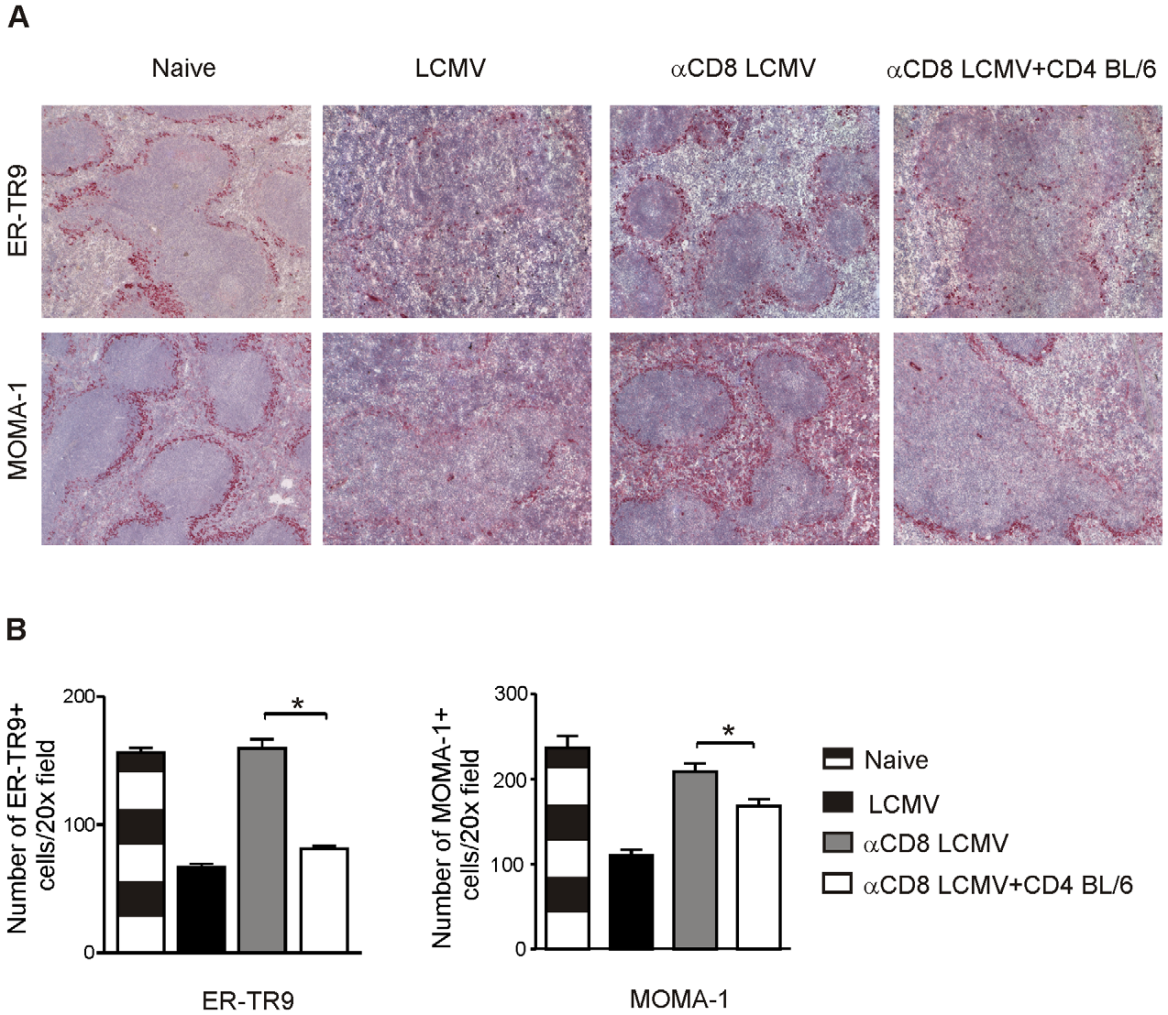
Immunohistochemistry analysis of the marginal zone. (**A,B**) BL/6 mice, BL/6 mice depleted of CD8^+^ T cells, and BL/6 mice depleted of CD8^+^ T cells receiving 1.5×10^7^ purified LCMV-immune CD4^+^ T cells were infected with LCMV. On day 11 after infection, spleen sections were stained for ER-TR9 (MZM) and MOMA-1 (MOMA) and the number of ER-TR9 and MOMA-1 positive cells was counted. For (A) one representative section of 3 mice per group is shown. For (B) number was counted in each staining in at least 5 microscopic visual fields (20× magnification) per mouse (n = 3). Data expressed as mean ± SEM (error bar).

Under the same experimental conditions, the integrity of the splenic marginal zone was analyzed by immunohistochemical staining of ER-TR9^+^ marginal zone macrophages (MZM) and MOMA-1^+^ (CD169^+^) marginal zone metallophilic macrophages (MOMA). The splenic marginal zone was largely destroyed in immunocompetent BL/6 mice 11 days after LCMV infection with reduced numbers of MZM and MOMA (Fig. 4A,B). In contrast, CD8-depleted BL/6 mice retained the marginal zone with numbers of MZM and MOMA comparable to naïve BL/6 mice (Fig. 4A,B). Interestingly, adoptive transfer of LCMV-immune CD4^+^ T cells to CD8-depleted BL/6 mice destroyed the marginal zone with reduced numbers of MZM and MOMA, comparable to LCMV-infected control BL/6 mice (Fig. 4A,B). Therefore, CD4^+^ T cells destroy lymphoid architecture in the absence of CD8^+^ T cells and their effect is focused to the marginal zone of the spleen.

### Analysis of B cell subtypes

**Figure 5:**
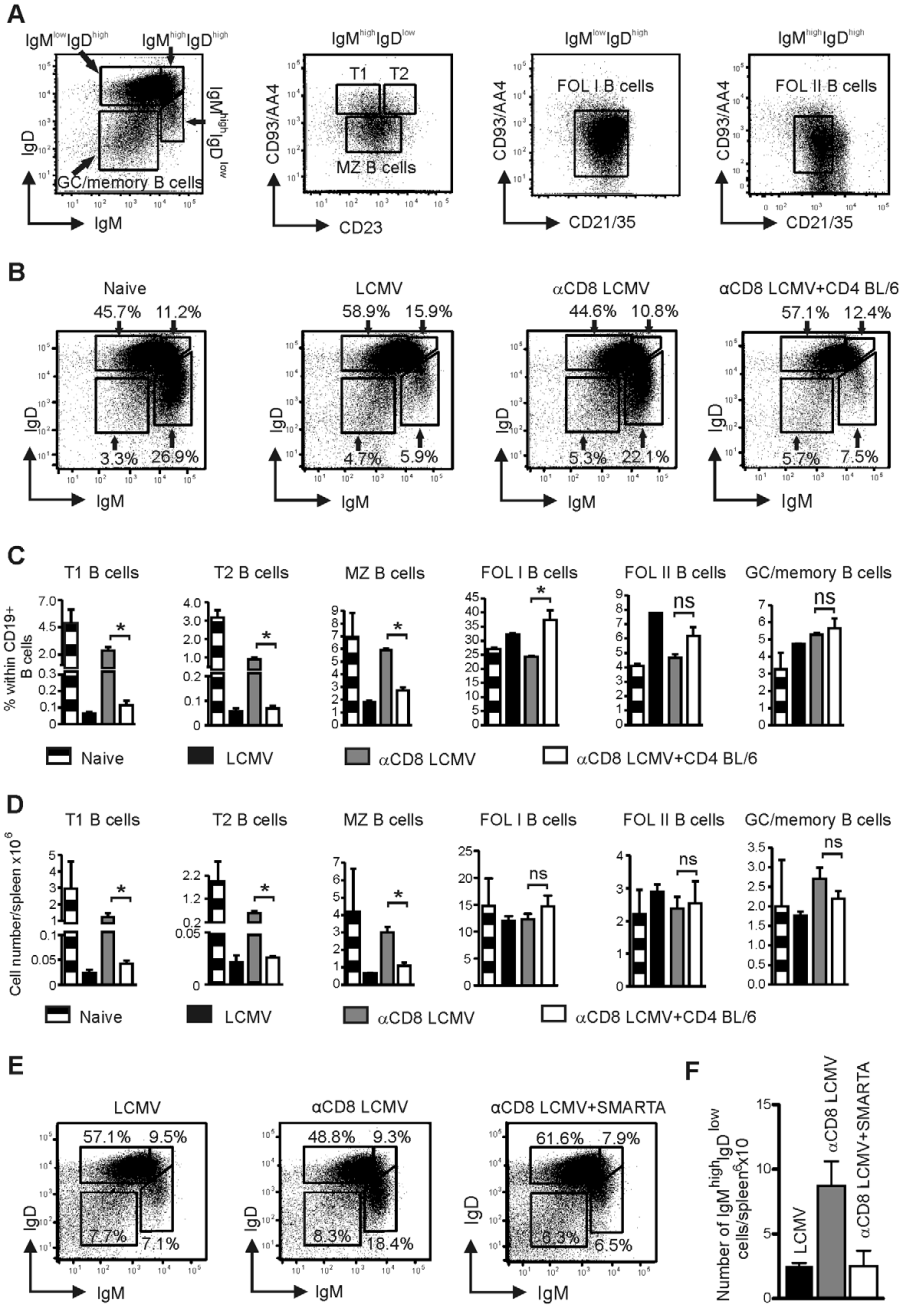
Analysis of the effect of CD4^+^ T cells on B cell subsets in spleen. (**A**) Example of splenic B cell subtype analysis. Single cell suspension of the spleen was first gated on lymphocytes by forward and side scatter and afterwards on CD19^+^ B cells. CD19^+^ B cells were divided in IgM^low^IgD^low^ GC/memory B cells, T1 B cells (CD19^+^IgM^high^IgD^low^CD93/AA4^+^CD23^−^), T2 B cells (CD19^+^IgM^high^IgD^low^CD93/AA4^+^ CD23^+^), MZ B cells (CD19^+^IgM^high^IgD^low^CD93/AA4^−^CD23^−^), FOL I B cells (CD19^+^IgM^low^IgD^high^CD21/35^int^), and FOL II B cells (CD19^+^IgM^high^IgD^high^CD93/AA4^−/low^CD21/35^int^) cells. (**B–D**) BL/6 mice, BL/6 mice depleted of CD8^+^ T cells and BL/6 mice depleted of CD8^+^ T cells receiving 1.5×10^7^ purified LCMV-immune CD4^+^ T cells were infected with LCMV. On day 11 after infection B cell subsets were analyzed as described in (A). (**B**) Analysis of IgM and IgD subtypes. Numbers indicate percentage (mean) within CD19^+^ B cells (n = 3 per group). (**C**) Percentage within CD19^+^ B cells and (**D**) absolute number of T1, T2, MZ, FOL I, and FOL II, and GC/memory B cells in the spleen. BL/6 naïve mice served as control. Mean ± SEM (error bars) of 3 animals per group is shown. For **B–D**, one representative experiment of 3 independent experiments is shown. (**E**, **F**) BL/6 mice, BL/6 mice depleted of CD8^+^ T cells and BL/6 mice depleted of CD8^+^ T cells receiving 1×10^7^ splenocytes of naïve SMARTA mice were infected with LCMV. On day 11 after infection, CD19^+^ splenocytes were analyzed for IgM and IgD subtypes. (**E**) Percentage (numbers indicate mean) within CD19^+^ B cells and (**F**) absolute number of IgM^low^IgD^high^, IgM^high^IgD^high^, IgM^high^IgD^low^ and GC/memory B cells were determined per spleen. Mean ± SEM (error bars) of 2–3 animals per group are shown.

We next wanted to define the effect of LCMV-specific CD4^+^ T cells on defined B cell subsets in the spleen. As described by Allman et al [18] and shown in Fig. 5A, splenic CD19^+^ B cells can be subdivided using multicolour flow cytometry into following groups: (i) IgM^high^IgD^low^ B cells, which contain transitional stage 1 (T1) B cells, transitional stage 2 (T2) B cells, and marginal zone (MZ) B cells, (ii) IgM^low^IgD^high^ B cells representing follicular type I (FOL I) B cells, and (iii) IgM^high^IgD^high^ B cells containing follicular type II (FOL II) B cells. IgM^low^IgD^low^ B cells contain germinal center (GC)/memory B cells [19]. Eleven days after infection, the frequency of IgM^high^IgD^low^ B cells was strongly reduced in LCMV-infected BL/6 mice when compared to naive controls (mean of 5.9% versus 26.9%; Fig. 5B). Accordingly, frequencies and absolute numbers of T1, T2, and MZ B cells were strongly reduced after LCMV infection (Fig. 5C–D). In contrast, frequencies and absolute numbers of B cells with an IgM^low^IgD^high^, IgM^high^IgD^low^ or IgM^high^IgD^high^ phenotype (FOL I, FOL II, and GC/memory B cells) remained similar or were increased (Fig. 5B–D). CD8-T cell depletion prevented the loss of B cells with IgM^high^IgD^low^ phenotype (mean frequency 22.1%, Fig. 5B), and frequencies and absolute numbers of T1, T2 and MZ B cells were similar to naïve BL/6 mice (Fig. 5C–D).

Interestingly, the transfer of LCMV-immune CD4^+^ T cells to CD8^+^ T cell-depleted and LCMV-infected mice drastically reduced the mean frequency of IgM^high^IgD^low^ B cells to 7.5% (Fig. 5B), a frequency similar to LCMV infected BL/6 mice. Accordingly, the transfer of LCMV-immune CD4^+^ T cells significantly reduced the frequency of T1, T2, and MZ B cells when compared to CD8-depleted BL/6 mice (Fig. 5C). Likewise, absolute numbers per spleen dropped from 1.2×10^6^ to 0.04×10^6^ for T1 B cells, from 0.46×10^6^ to 0.02×10^6^ for T2 B cells and from 3.0×10^6^ to 1.1×10^6^ for MZ B cells after adoptive transfer of LCMV-immune CD4^+^ T cells. In contrast, B cells with other IgMIgD phenotypes (FOL I, FOL II, and GC/memory B cells) remained unchanged or were increased after transfer of LCMV-immune CD4^+^ T cells to CD8-depleted mice.

To analyze if the same effect also occurred after adoptive transfer of a high frequency of naïve LCMV-specific CD4^+^ T cells, we replaced LCMV-immune CD4^+^ T cells by naïve CD4^+^ T cells from SMARTA mice. The adoptive transfer of SMARTA cells to CD8-depleted mice reduced the mean frequency of IgM^high^IgD^low^ B cells from 18.4% to 6.5% and the mean absolute number from 8.7×10^6^ to 2.5×10^6^/spleen (Fig. 5E,F). In contrast, B cell subgroups with a different IgMIgD phenotype remained unchanged or were increased (Fig. 5E). In summary, these data indicate, that CD4^+^ T cells selectively reduce IgM^high^IgD^low^ B cells during LCMV infection.

### Mechanism of IgM^high^IgD^low^ B cell elimination by cytotoxic CD4^+^ T cells

**Figure 6:**
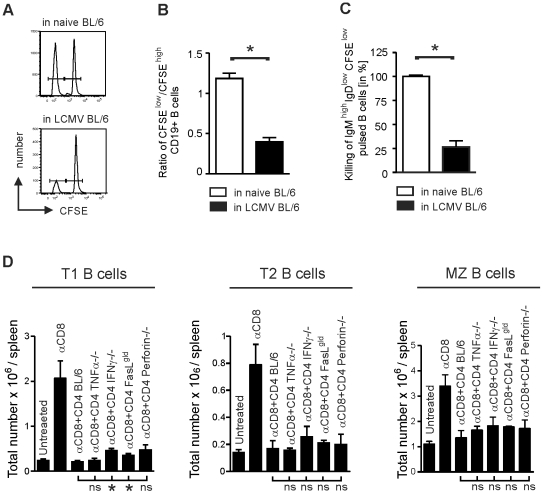
Effector mechanisms of CD4^+^ T cell-mediated immunopathology. (**A**) Naïve B cells were pulsed with LCMV GP61 peptide and labelled with a low concentration of CFSE (CFSE^low^). Non-peptide pulsed naïve B cells were used as controls and labelled with a 10-times higher concentration of CFSE (CFSE^high^). CFSE^low^ and CFSE^high^ B cells were injected in a ratio of 1:1 to LCMV infected (day 11) BL/6 mice or naïve BL/6 mice and analyzed 18 hours later in the spleen by flow cytometry. (**A**) Representative example of each group. (**B**) The ratio of CFSE^low^/CFSE^high^ CD19^+^ B cells was calculated. (**C**) Percentage of IgM^high^IgD^low^ B cells within CD19^+^ B cells was determined. Values of naïve BL/6 mice were taken as 100%. (**D**) BL/6 mice, BL/6 mice depleted of CD8^+^ T cells, and BL/6 mice depleted of CD8^+^ T cells receiving 1.8×10^5^ LCMV-GP61-restricted IFNγ-producing splenocytes of BL/6, TNFα^−/−^, FasL*^gld^* or perforin^−/−^ mice or 1.8×10^5^ LCMV-GP61-restricted TNFα-producing splenocytes of IFNγ^−/−^ mice were infected with LCMV. Eleven days after infection the absolute number of T1, T2 and MZ B cells per spleen was analyzed in the spleen. Mean ± SEM (error bars) is shown (n = 3). Statistical analysis was calculated between mice receiving CD4^+^ T cells from BL/6 mice and mice receiving CD4^+^ T cells from TNFα^−/−^, IFNγ^−/−^, FasL*^gld^* or perforin^−/−^ mice.

To examine if CD4^+^ T cells can directly kill IgM^high^IgD^low^ B cells, naïve B cells were labelled with a low CFSE concentration (CFSE^low^) and pulsed with LCMV GP61 peptide, a MHC-class II restricted epitope. As a control, non-peptide pulsed naïve B cells were labelled with a 10-fold higher concentration of CFSE (CFSE^high^). CFSE^low^ and CFSE^high^ B cells were injected in a ratio of 1:1 to LCMV infected BL/6 mice (11 days after infection) or naïve BL/6 mice. Peptide-specific killing was assessed 18 hours later and revealed that more than half of LCMV-GP61 pulsed CD19^+^ B cells were eliminated (Fig. 6A,B). Elimination after adoptive transfer of CD4^+^ T cells included all B cell subtypes and, compared to naïve BL/6 mice, IgM^high^IgD^low^ B cells in LCMV infected mice were reduced by approximately 70% (Fig. 6C). These data indicate that LCMV-GP61 presenting IgM^high^IgD^low^ B cells are a direct target of cytotoxic CD4^+^ T cells.

To understand the mechanism of IgM^high^IgD^low^ B cell reduction by CD4^+^ T cells, LCMV-immune CD4^+^ T cells from mice lacking important effector cytokines and cytolytic pathways such as IFNγ, TNFα, perforin and FasL (gld) were generated for adoptive transfer experiments. Because virus control is different in all these knockout mice, purified CD8^+^ T cells (2×10^7^ cells) of naïve immunocompetent BL/6 mice were adoptively transferred to all mice before LCMV inoculation. Seventeen days later, LCMV was not detectable in peripheral blood by conventional *in vitro* focus forming assay in all mice (Fig. S2) and CD4^+^ T cells produced the effector cytokines IFNγ and/or TNFα after restimulation with GP61 (Fig. S3). Splenocytes of these mice were then purified for CD4^+^ T cells by MACS and the number of CD4^+^ T cells containing 1.8×10^5^ IFNγ-producing LCMV GP61-specific CD4^+^ T cells from TNFα^−/−^, perforin^−/−^, FasL*^gld^* and BL/6 mice were adoptively transferred to groups of naïve CD8-depleted BL/6 mice. The number of CD4^+^ T cells from IFNγ^−/−^ mice was adjusted for TNFα production. On day 11 after LCMV infection, B cell subgroups were analyzed as before by flow cytometry. T1, T2 and MZ B cells were clearly reduced after transfer of CD4^+^ T cells from TNFα^−/−^, IFNγ^−/−^, perforin^−/−^, or FasL*^gld^* mice (Fig. 6D). There was a statistically significant elevated number of T1 B cells in CD8-depleted mice treated with CD4^+^ T cells lacking IFNγ or FasL in comparison to CD8-depleted mice receiving control CD4^+^ T cells from BL/6 mice. However, quantitatively this difference was only minimal. For T2 and MZ B cells no difference could be found between the different groups of adoptively transferred CD4^+^ T cells.

### CD4^+^ T cell mediated immunopathology in the liver

**Figure 7:**
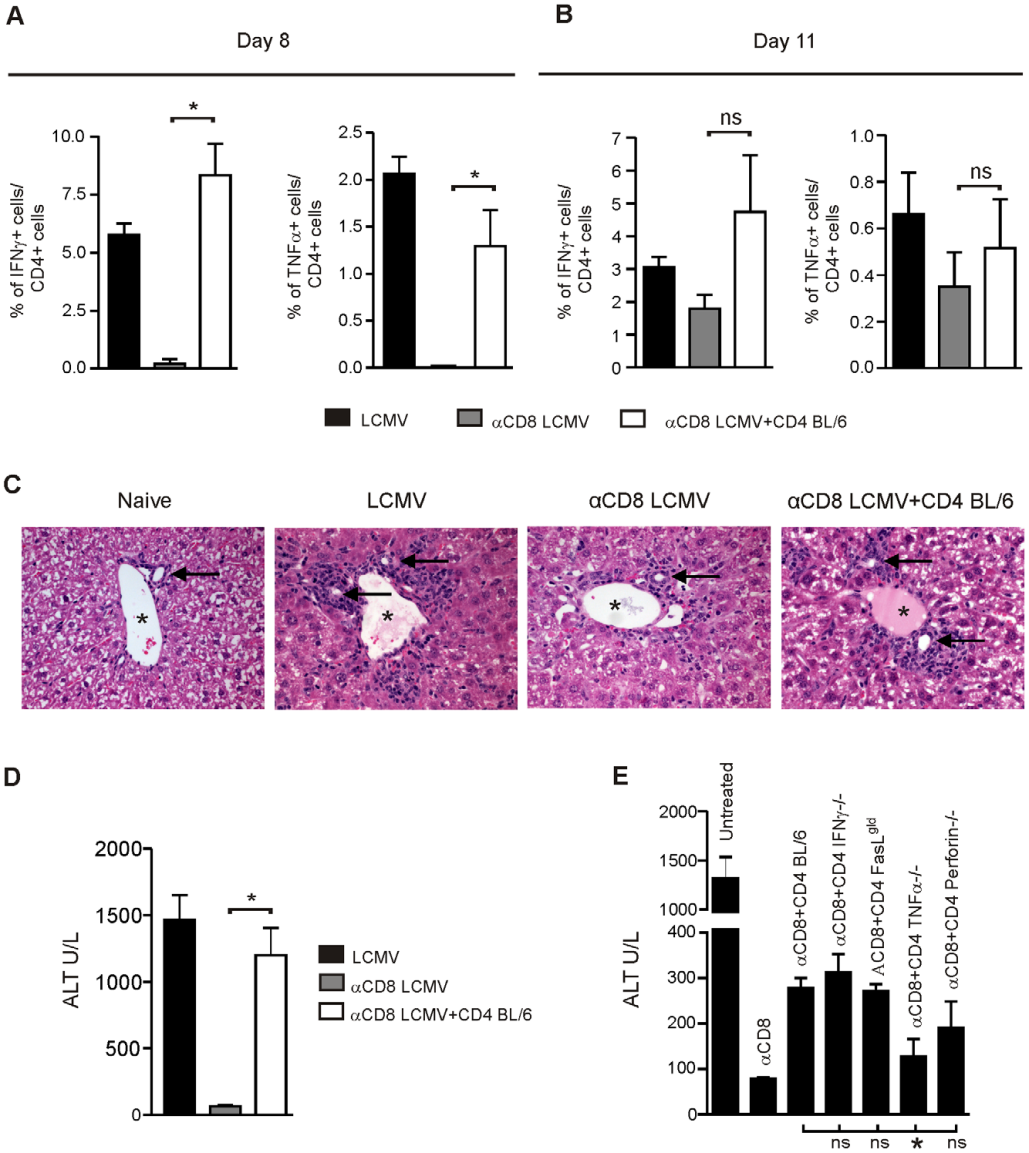
CD4^+^ T cell-mediated destruction of liver tissue. (**A–D**) BL/6 mice, BL/6 mice depleted of CD8^+^ T cells and BL/6 mice depleted of CD8^+^ T cells receiving 1.5×10^7^ purified LCMV-immune CD4^+^ T cells i.v. at day 0, were all infected with LCMV. On day 8 (**A**) and 11 (**B**) after infection, CD4^+^ lymphocytes isolated from the liver were restimulated in vitro for 5 h with GP61 and analyzed for intracellular IFNγ and TNFα production by flow cytometry. (**C**) On day 11 after infection, liver sections (H&E staining) were analyzed. One representative example of 2–3 mice per group is shown. Image shows portal tract. Asterix: portal vein. Arrow: bile duct. (**D**) Level of ALT was measured in the serum. (**E**) BL/6 mice, BL/6 mice depleted of CD8^+^ T cells, and BL/6 mice depleted of CD8^+^ T cells receiving 1.8×10^5^ LCMV-GP61-restricted IFNγ-producing splenocytes of BL/6, TNFα^−/−^, FasL*^gld^* or perforin^−/−^ mice or 1.8×10^5^ LCMV-GP61-restricted TNFα-producing splenocytes of IFNγ^−/−^ mice were infected with LCMV. Eleven days after infection, level of ALT was measured in the serum. For (A,B,D,E) mean ± SEM of three mice per group are shown. Statistical analysis was calculated between mice receiving CD4^+^ T cells from BL/6 mice and mice receiving CD4^+^ T cells from TNFα^−/−^, IFNγ^−/−^, FasL*^gld^* or perforin^−/−^ mice.

Similar to the immune responses analyzed in the spleen, IFNγ and TNFα production by LCMV-specific CD4^+^ T cells was reduced or even absent in the liver of CD8-depleted mice on day 8 and 11 after infection (Fig. 7A,B). Again, the frequency of IFNγ- and TNFα-producing LCMV-specific CD4^+^ T cells was restored after transfer of purified LCMV-immune CD4^+^ T cells (Fig. 7A,B). Histological analysis of the liver 11 days after LCMV infection showed moderate portal-periportal and intralobular mononuclear inflammation in immuncompetent BL/6 mice (Fig. 7C). In CD8-depleted LCMV-infected BL/6 mice, mononuclear inflammation was minimal and restricted to the portal tract with no extension to the periportal or intralobular region. In contrast, adoptive transfer of LCMV-immune CD4^+^ T cells resulted in a low to moderate portal-periportal and intralobular mononuclear inflammation.

Elevated levels of serum alanin-aminotransferase (ALT) is an indicator of liver cell damage. Eleven days after infection, control BL/6 mice but not CD8-depleted BL/6 mice had elevated ALT concentrations in the serum (Fig. 7D). The transfer of purified LCMV-immune CD4^+^ T cells to CD8-depleted LCMV infected BL/6 recipients resulted in elevated ALT levels (Fig. 7D). Thus, CD4^+^ T cells are able to induce liver cell damage during LCMV infection. A potential role of CD4^+^ T cells in causing hepatitis has already been shown earlier [20], however the mechanism has never been elucidated. Therefore, we analyzed ALT concentrations in the sera of CD8-depleted LCMV-infected BL/6 mice, receiving LCMV-immune CD4^+^ T cells from mice deficient for important effector cytokine and cytolytic pathways as described before (see Fig. 6C). The transfer of CD4^+^ T cells from IFNγ^−/−^, FasL*^gld^* and perforin^−/−^ mice but not from TNFα^−/−^ mice resulted in a significant increase in serum ALT levels (Fig. 7E). Of note, the magnitude of the increase in serum ALT levels is directly related to the number of adoptively transferred LCMV-immune CD4^+^ T cells. The lower number of transferred CD4^+^ T cells from BL/6 mice (1.1×10^7^/mouse) in the experiment shown in Fig. 7E explains the relatively moderate increase of serum ALT levels when compared to figure 7D, where 1.5×10^7^ CD4^+^ T cells were adoptively transferred.

## Discussion

Infection with LCMV causes T cell mediated immunopathology. Dependent on the dose and tropism of the virus, this results in destruction of lymphoid organ architecture, choriomeningitis, and/or hepatitis [1], [2]. Destruction of lymphoid organ architecture was mainly attributed to CD8^+^ T cells, because it was prevented by CD8-depletion [1]. However, in the absence of CD8^+^ T cells, LCMV persists at high titers and leads to functional inactivation and exhaustion of CD4^+^ T cells [13]. Therefore, the role of CD4^+^ T cells in the destruction of lymphoid architecture has never been investigated so far. To avoid functional inactivation of LCMV-specific CD4^+^ T cells, we used two different approaches. We adoptively transferred either purified LCMV-immune CD4^+^ T cells or naïve LCMV-GP61 specific (SMARTA) CD4^+^ T cells to CD8-depleted mice. These experiments clearly revealed that CD4^+^ T cells can induce immunopathology in spleen and liver. LCMV-immune CD4^+^ T cells that are transferred to naïve BL/6 mice and rechallenged with LCMV may behave differently than endogenous activated CD4^+^ T cells. Therefore, we repeated the experiments with adoptive transfer of naïve SMARTA CD4^+^ T cells. The immunopathological sequel after transfer of naïve SMARTA CD4^+^ T cells was comparable to LCMV-immune polyclonal CD4^+^ T cells. This suggests that the persistence of functional CD4^+^ T cells in CD8^+^ T cell depleted hosts is rather a consequence of the increased LCMV-specific CD4^+^ T cell precursor frequency than the activation status.

Interestingly, CD4^+^ T cells selectively destroyed the marginal zone, whereas complete disruption of B- and T cell regions only occurred in CD8-competent control mice. This indicates that part of the immunopathological sequel, such as the destruction of the splenic marginal zone, can be mediated by CD4^+^ T cells, whereas others depend on the presence of functional CD8^+^ T cells. Accordingly, functional CD4^+^ T cells reduced IgM^high^IgD^low^ T1, T2 and MZ B cells, whereas B cells with a different IgM/IgD phenotype (FOL I, FOL II, GC/memory B cells) remained largely stable. Whereas little is known about the role of T1 and T2 B cells in immune response, it is well documented that MZ B cells are crucial for the induction of T cell-independent and T cell-dependent antibody responses [21], [22], [23]. Therefore, the observed reduction of T cell dependent LCMV-specific neutralizing antibodies after transfer of CD4^+^ T cells may be a result of reduced MZ B cells. In agreement with this hypothesis, mice lacking MZ B cells showed a reduced IgG antibody response against T cell-dependent antigens such as *Borrelia burgdorferi* [24]. In addition, (CD169^+^) marginal zone metallophilic macrophages (MOMA) are essential for the initiation of antiviral B cell response [25]. Since MOMA are also reduced by CD4^+^ T cells, this may further contribute to the reduction in neutralizing antibody titers. A correlation of CD4^+^ T cell activity with reduced and delayed neutralizing antibody responses to LCMV has already been reported earlier [26]. This was attributed to competition on survival factors or anatomical niches due to a CD4^+^ T cell induced polyclonal B cell activation and hypergammaglobulinemia. However, our earlier experiments showed that higher titers of LCMV-neutralizing antibodies were produced in CD27-deficient mice than in BL/6 mice despite of higher gammaglobulin levels in CD27-deficient mice [16].

The integrity of the splenic marginal zone is dependent on several chemokines. Marginal zone macrophage localization is regulated by CCL19 and CCL21 [27] and activated marginal zone B cells are attracted by CXCL13 [22]. CD8-depleted BL/6 mice had a strongly reduced expression of CCL21, CCL19 and CXCL13. However, CD4^+^ T cells did not further affect the expression of these chemokines. This is in accordance with our results that stromal cells (FDCs and FRCs) producing these chemokines were not reduced in numbers after transfer of CD4^+^ T cells. Together, this indicates that CD4^+^ T cell mediated destruction of the splenic marginal zone is not a result of changes in chemokine expression. Our experiments with peptide pulsed and CFSE labelled B cells rather indicate that MZ B cells are eliminated directly by cytotoxic CD4^+^ T cells. By virtue of their abundant expression of MHCII and costimulatory molecules, MZ B cells are efficient antigen presenting cells for CD4^+^ T cells [23]. While presenting LCMV-specific antigens to CD4^+^ T cells, MZ B cells probably become a direct target of activated CD4^+^ T cells. Interestingly, CD4^+^ T cell mediated elimination of MZ B cells did not reduce virus titers in the spleen. This is in accordance with current evidence that B cells are not infected directly by LCMV but capture and present exogenous antigen [28]. Similarly, MOMA may also be a direct target of cytotoxic CD4^+^ T cells, as they constitute a reservoir for LCMV replication [29] and express MHCII molecules [25]. However, resident cells of the spleen depend on each other for their localization and maintenance [3]. Therefore, from our results we cannot define which cell populations of the splenic marginal zone are direct targets of cytotoxic CD4^+^ T cells and which cell populations are reduced indirectly as a consequence of the loss of the integrity of the marginal zone.

Cytotoxic CD4^+^ T cells have been reported after infection with various pathogens and several mechanisms of cytotoxicity have been described. These include i) Fas/FasL in EBV [30]- and LCMV [31]-infection, ii) perforin in influenza [32], EBV [33] and HIV [34] infection and iii) IFNγ in gammaherpesvirus 68 infection [35]. We could not define a single pathway responsible for CD4^+^ T cell mediated reduction of IgM^high^IgD^low^ B cells in our experimental set-up. Although T1 B cell lysis seemed to depend in part on IFNγ and Fas-FasL interaction, the difference to the control group was small, indicating that each of this single effector pathway by its own is of minor physiological importance. In contrast, an earlier publication showed that B cells were killed after LCMV infection by CD4^+^ T cells in a Fas-Fas ligand mechanism [31]. However, that study analyzed adoptively transferred naive B cells labelled with GP61 peptide, which can not directly be compared to our study analyzing activated B cells in the course of a LCMV infection.

Liver enzyme levels after LCMV infection are not elevated in CD8-depleted BL/6 mice suggesting that CD8^+^ T cells are the only cells responsible for liver cell damage. However, our experiments suggest that CD4^+^ T cells also play a role in the induction of hepatitis. As hepatocytes are infected by LCMV and upregulate MHCII molecules after LCMV infection, ours and earlier experiments [20] indicate that they may become a direct target of cytotoxic CD4^+^ T cells. However, as for the spleen, we cannot exclude that secondary effects are involved in liver cell damage, e.g. activation of Kupffer cells, macrophages, or of hepatotoxic NK/NKT cells by CD4^+^ T cells [36], [37]. Our experiments revealed that the induction of hepatitis by CD4^+^ T cells depended at least in part on TNFα. Therefore, CD4^+^ T cells are involved in immunopathological sequel in various organs, but the relative importance of the cytolytic pathways may depend on the organ analyzed.

In several widespread human pathogens such as *Plasmodium falciparum* (malaria) [6], *Leishmania donovani* [8], Lassa virus [7] and HIV [5], [38] lymphoid architecture is destroyed and consequently the host's immune system is suppressed. Further studies will be necessary to evaluate the contribution of CD4^+^ T cells in immunopathology in these infections, especially in viral infections that are characterized by a late and inefficient induction of neutralizing Abs, such as HIV and HCV. As a parallel to LCMV, a reduction of marginal zone B cells has been documented in HIV infection [39] and interestingly, early hyperactivity of CD4^+^ T cells is a risk factor for disease progression after HIV infection [40]. Likewise, a strong T helper cell response was associated with low neutralizing antibodies after HCV infection [41]. Therefore, understanding the mechanisms of lymphoid destruction in different infections may help to design novel therapies.

## Materials and Methods

### Ethics statement

Animal experiments were carried out in strict accordance with the regulations of the Veterinary office of the Canton Bern (Switzerland) and the Swiss Animal Protection Law. The protocol was approved (Permit Number: 84-08) by the Veterinary office of the Canton Bern.

### Mice

C57BL/6 (BL/6) mice were purchased from Harlan (Amsterdam, Netherlands). SMARTA transgenic mice, IFNγ^−/−^ and Perforin^−/−^ mice were obtained from the Institute for Laboratory Animals (Zurich, Switzerland). TNFα^−/−^ mice were from C. Müller (Bern, Switzerland) and FasL-deficient FasL*^gld^* mice from N. Corazza (Bern, Switzerland).

### Viruses and peptide

LCMV-WE was from R. M. Zinkernagel (Zurich, Switzerland) and was propagated on L929 fibroblast cells. If not indicated differently 10^6^ pfu LCMV-WE was used for all experiments. The LCMV glycoprotein peptide amino acid GP61 (GLNGPDIYKGVYQFKSVEFD) was purchased from NeoMPS SA (Strasbourg, France).

### Detection of virus and neutralizing antibody titers

The detection of LCMV titer and neutralizing antibodies has been described earlier [42].

### Immunofluorescence and immunohistochemistry

Tissues were embedded in optimum cutting temperature compound (O.C.T medium, Tissue-Tek, Sakura) without prior fixation and snap-frozen. For immunofluorescence, cryostat sections (8 µm in thickness) were stained as previously described [43], and acquired on an Axioplan microscope with AxioCam MRm (Zeiss) or on a DM IRE2 microscope with a laser-scanning confocal head TCS SP2 acousto-optical beam splitter (Leica). For immunohistochemistry, cryostat sections of 5-µm thickness were treated as described [44] and stained with rat monoclonal antibodies against metallophilic marginal zone macrophages (MOMA-1; BMA Biomedicals AG, Augst, Switzerland) and ER-TR9 [45].

### CD8^+^ T cell depletion

Mice were treated intraperitoneally (i.p.) with 200 µg of αCD8 mAb (YTS169.4, bioXcell, West Lebanon, NH) on days 0, 1, 3, 5, 7 and 10 after adoptive transfer of CD4^+^ T cells and LCMV infection. Efficiency of depletion was verified by flow cytometry analysis.

### Antibodies and flow cytometry

αCD4-PE, αCD4-PE-Cy5, αCD19-FITC, αCD19-APC-Cy7, αIgD-PE, αIgM-PE-Cy7, αCD93/AA4-APC, αCD23-Biotin, αCD21/35-FITC, αIFN-γ-FITC, αTNFα-PE, were purchased from eBioscience (San Diego, CA). For intracellular staining, lymphocytes (10^6^/wells) were stimulated with GP61 peptide (at 10^−6^ M per well) in the presence of recombinant IL-2 (25 U/ml, ProSpec, Rehovot, Israel) and 5 mg/ml Brefeldin A (Sigma-Aldrich, Switzerland) for 5 hours. Cells were stained for surface molecules, fixed with 4% paraformaldehyde in PBS, and then cell membranes were permeabilized with Perm-Buffer (PBS, 2% FCS, 5 mM EDTA, 0.1% saponin, 0.2% NaN_3_) and stained with αIFNγ-FITC or αTNFα-PE. Relative fluorescence intensities were measured with a FACScan™ or BD™ LSRII (BD, Mountain View, CA) and analyzed using FlowJo™ software (Tree Star, Ashland, OR).

### Purification of LCMV-immune CD4^+^ T cells

BL/6 mice were infected with 200 pfu LCMV-WE. 17 days later splenocytes were harvested and enriched for CD4^+^ T cells by positive selection with MACS (Miltenyi Biotec, Bergisch Gladbach, Germany) and were adoptively transferred i.v. to recipient mice. The purity of CD4^+^ T cells was ≥90%.

### Isolation of hepatic lymphocytes

To isolate hepatic lymphocytes, liver was removed, and cut in small pieces. Afterwards liver was digested with BSS containing 2% FCS, 0.6% BSA, 5 mM CaCl^2^, 5 mM MgCl^2^, 1 mg/ml collagenase Type IA (Sigma-Aldrich), 20 µg/ml DNase I (Roche) for 15 min at 37°C. In a next step, liver was filtered through a sterile 40-µm nylon cell strainer (BD Biosciences) and washed once with BSS before erythrocytes were removed by Puregene red blood cell lysis solution (Qiagen). Finally, lymphocytes were isolated by Ficoll gradient centrifugation (Ficoll-Paque^Plus^, Amersham Biosciences, Uppsala, Sweden).

### Real time RT PCR of chemokines

Spleens were shock frozen and disrupted in Trizol. RNA was isolated according to manufacture guidelines (Invitrogen) and real time PCR analysis was performed using QuantiFast SYBR Green (Eurogentech) PCR kit. The following primer combination was used: GAPDH forward 5′ CCA CCC CAG CAA GGA GAC T and GAPDH reverse 5′ GAA ATT GTG AGG GAG ATG CT; Ccl19-atg 5′ -CTG CCT CAG ATT ATC TGC CAT- 3′ and 5′ -AGG TAG CGG AAG GCT TTC AC- 3; Ccl21-ser 5′ -ATC CCG GCA ATC CTG TTC TC- 3′ and 5′ -GGT TCT GCA CCC AGC CTT C- 3′; CXCL13 5′- GAG GCT CAG CAC AGC AAC-3′ and 5′-TTG AAA TCA CTC CAG AAC ACC TAC A- 3′.

### Alanine aminotransferase

We measured ALT with a serum multiple biochemical analyzer (cobas® 8000 modular analyser, Roche, Switzerland).

### Statistical evaluation

For statistical analysis Graph Pad Prism Version 5 (GraphPad Software) was used. Significances were tested using an unpaired, two-tailed student's t-test. Data was considered statistically significant when p-value was <0.05. Symbol * means p<0.05, ns =  not significant.

## Supporting Information

Figure S1 **Immunofluorescence analysis of CXCL13 and CCL21 protein expression.** (**A**) BL/6 mice, BL/6 mice depleted of CD8^+^ T cells, and BL/6 mice depleted of CD8^+^ T cells receiving 1.5×10^7^ purified LCMV-immune CD4^+^ T cells were infected with LCMV. On day 11 after infection spleen sections were stained for CXCL13 protein (red), and Laminin (blue), or CCL21 protein (green). TZ: T cell zone, BZ: B cell zone. Arrows show sites of CXCL13 expression co-localizing frequently with laminin-expressing cells in LCMV-exposed spleens. Large dots in the red pulp are artifactual staining of cells expressing endogenous HRP. One representative section of 3 mice per group is shown. BL/6 naïve mice served as control. Size bar represents 100μm.
(TIF)

Figure S2 **Virus control in mice deficient in T cell effector mechanisms.** 2×10^7^ CD8^+^ T cells from naïve BL/6 mice were adoptively transferred to IFNγ^-/-^, TNFα^-/-^, perforin^-/-^ and FasL*^gld^* deficient mice before infection with 200 pfu LCMV. Seventeen days after infection, virus titer was measured by plaque forming assay of the peripheral blood from all mice. Each symbol represents one mouse. As an assay control LCM virus with a titer of 3×10^7^pfu/ml was taken.
(TIF)

Figure S3 **IFNγ and TNFα production in mice deficient in T cell effector mechanisms.** 2×10^7^ CD8^+^ T cells from naïve BL/6 mice were adoptively transferred to IFNγ^-/-^, TNFα^-/-^, perforin^-/-^ and FasL*^gld^* deficient mice before infection with 200 pfu LCMV. Seventeen days after infection, CD4^+^ T lymphocytes from the spleen were restimulated *in vitro* for 5h with p13 and analyzed for intracellular IFNγ and TNFα production by flow cytometry. Numbers indicate percentage (mean, n = 3) of CD4^+^ T cells producing IFNγ and TNFα.
(TIF)
