# Supplementary figures and images for: Destruction of Lymphoid Organ Architecture and Hepatitis Caused by CD4+ T Cells
Source: PLoS One. 2011 Sep 23;6(9):e24772. doi: 10.1371/journal.pone.0024772 (PMC3179489; doi:10.1371/journal.pone.0024772)

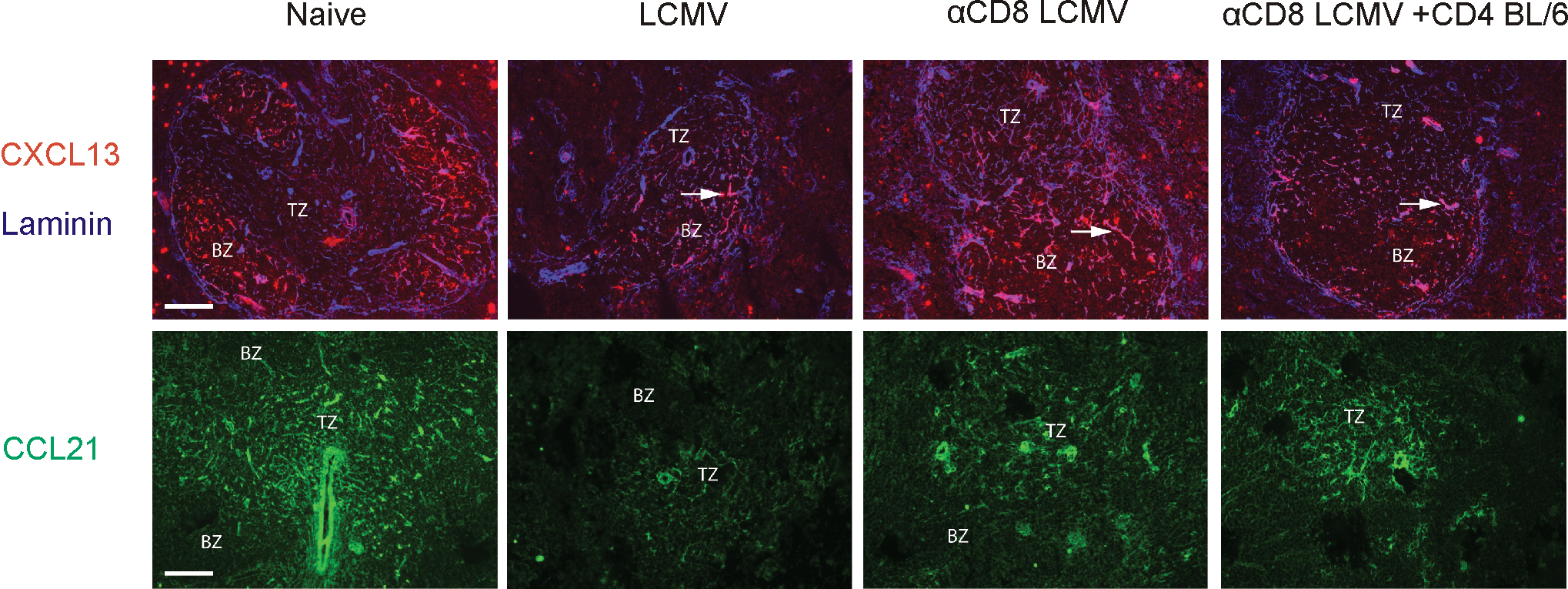

Supplement: Figure S1 — Immunofluorescence analysis of CXCL13 and CCL21 protein expression. (A) BL/6 mice, BL/6 mice depleted of CD8+ T cells, and BL/6 mice depleted of CD8+ T cells receiving 1.5×10^7^ purified LCMV-immune CD4+ T cells were infected with LCMV. On day 11 after infection spleen sections were stained for CXCL13 protein (red), and Laminin (blue), or CCL21 protein (green). TZ: T cell zone, BZ: B cell zone. Arrows show sites of CXCL13 expression co-localizing frequently with laminin-expressing cells in LCMV-exposed spleens. Large dots in the red pulp are artifactual staining of cells expressing endogenous HRP. One representative section of 3 mice per group is shown. BL/6 naïve mice served as control. Size bar represents 100μm. (TIF) [file pone.0024772.s001.tif]

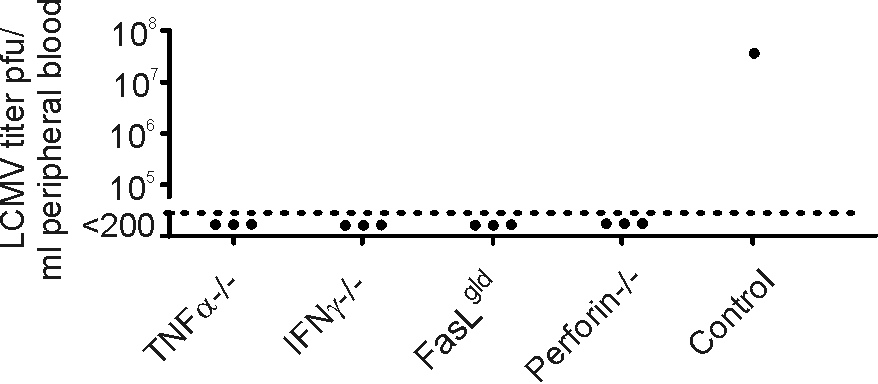

Supplement: Figure S2 — Virus control in mice deficient in T cell effector mechanisms. 2×10^7^ CD8+ T cells from naïve BL/6 mice were adoptively transferred to IFNγ-/-, TNFα-/-, perforin-/- and FasLgld deficient mice before infection with 200 pfu LCMV. Seventeen days after infection, virus titer was measured by plaque forming assay of the peripheral blood from all mice. Each symbol represents one mouse. As an assay control LCM virus with a titer of 3×10^7^pfu/ml was taken. (TIF) [file pone.0024772.s002.tif]

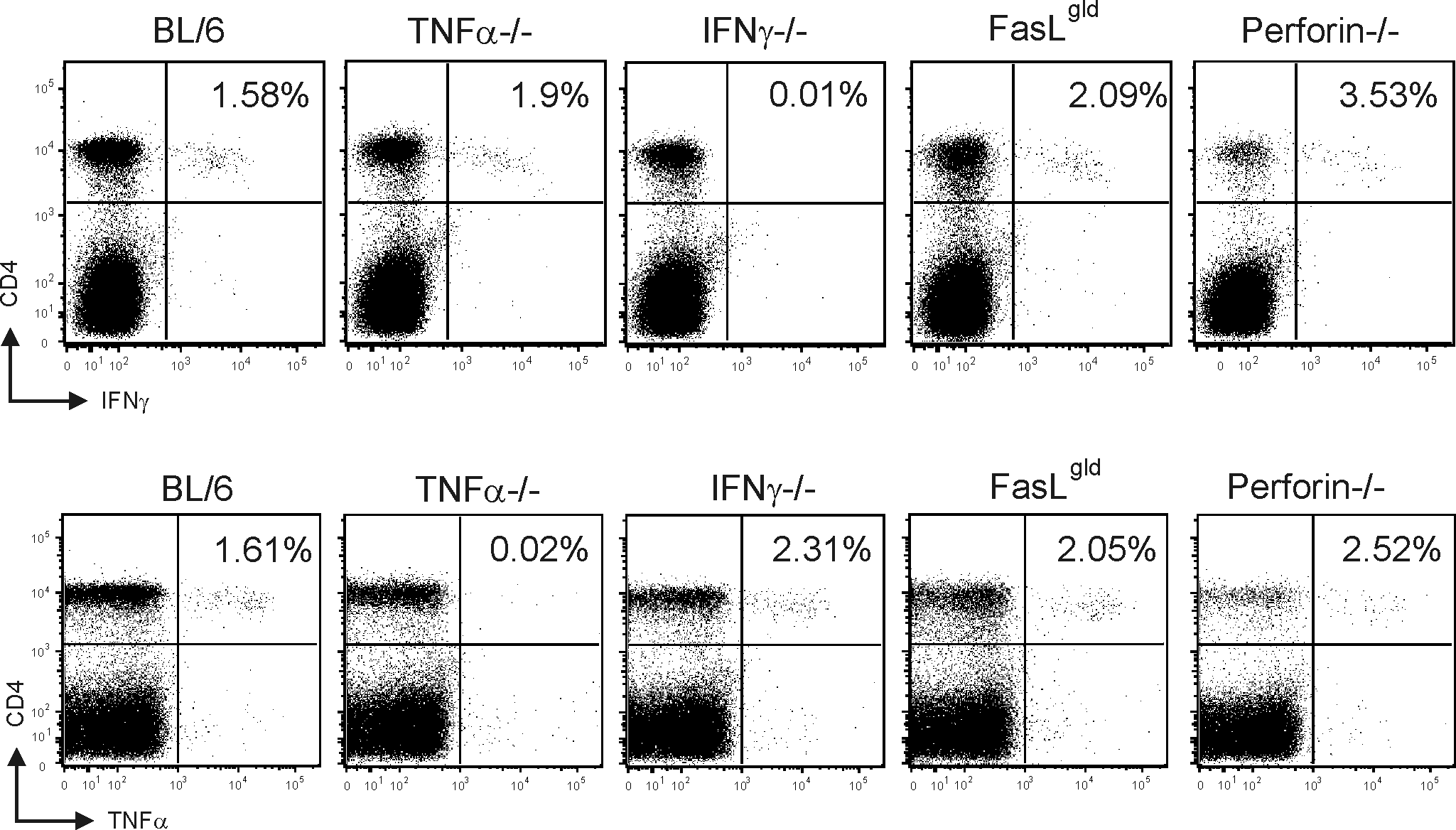

Supplement: Figure S3 — IFNγ and TNFα production in mice deficient in T cell effector mechanisms. 2×10^7^ CD8+ T cells from naïve BL/6 mice were adoptively transferred to IFNγ-/-, TNFα-/-, perforin-/- and FasLgld deficient mice before infection with 200 pfu LCMV. Seventeen days after infection, CD4+ T lymphocytes from the spleen were restimulated in vitro for 5h with p13 and analyzed for intracellular IFNγ and TNFα production by flow cytometry. Numbers indicate percentage (mean, n = 3) of CD4+ T cells producing IFNγ and TNFα. (TIF) [file pone.0024772.s003.tif]
